# Oldest Near-Complete Acanthodian: The First Vertebrate from the Silurian Bertie Formation Konservat-Lagerstätte, Ontario

**DOI:** 10.1371/journal.pone.0104171

**Published:** 2014-08-05

**Authors:** Carole J. Burrow, David Rudkin

**Affiliations:** 1 Ancient Environments, Queensland Museum, Brisbane, Queensland, Australia; 2 Department of Natural History – Palaeobiology, Royal Ontario Museum, Toronto, Ontario, Canada; Laboratoire Arago, France

## Abstract

**Background:**

The relationships between early jawed vertebrates have been much debated, with cladistic analyses yielding little consensus on the position (or positions) of acanthodians with respect to other groups. Whereas one recent analysis showed various acanthodians (classically known as ‘spiny sharks’) as stem osteichthyans (bony fishes) and others as stem chondrichthyans, another shows the acanthodians as a paraphyletic group of stem chondrichthyans, and the latest analysis shows acanthodians as the monophyletic sister group of the Chondrichthyes.

**Methodology/Principal Findings:**

A small specimen of the ischnacanthiform acanthodian *Nerepisacanthus denisoni* is the first vertebrate fossil collected from the Late Silurian Bertie Formation Konservat-Lagerstätte of southern Ontario, Canada, a deposit well-known for its spectacular eurypterid fossils. The fish is the only near complete acanthodian from pre-Devonian strata worldwide, and confirms that *Nerepisacanthus* has dentigerous jaw bones, body scales with superposed crown growth zones formed of ondontocytic mesodentine, and a patch of chondrichthyan-like scales posterior to the jaw joint.

**Conclusions/Significance:**

The combination of features found in *Nerepisacanthus* supports the hypothesis that acanthodians could be a group, or even a clade, on the chondrichthyan stem. Cladistic analyses of early jawed vertebrates incorporating *Nerepisacanthus*, and updated data on other acanthodians based on publications in press, should help clarify their relationships.

## Introduction

The group of early jawed fishes traditionally referred to the Acanthodii is emerging as pivotal in our understanding of relationships between extinct and extant gnathostomes, i.e. jawed fishes [1], [2]. However, the oldest history of the Acanthodii is poorly known, because most species described from the early Paleozoic Era (before the Devonian Period) are only based on isolated elements including scales, fin spines, tooth whorls, and dentigerous jaw bones [3]. Few taxa are known from articulated body fossils [4]. All specimens of one such rare species, the ischnacanthiform *Onchus graptolitarum* [5] from the Late Silurian (Pridoli) of the Czech Republic – to date, the only Silurian acanthodian represented by several partial articulated fish – have been long lost. The holotype specimen of the only other undisputed Silurian acanthodian taxon based on an articulated fossil, *Nerepisacanthus denisoni* [4] from the Ludlow or Pridoli of New Brunswick, Canada, lacks most of the head and the posterior half of the body, with the anterior dorsal spine being the only fin spine preserved.

**Figure 1:**
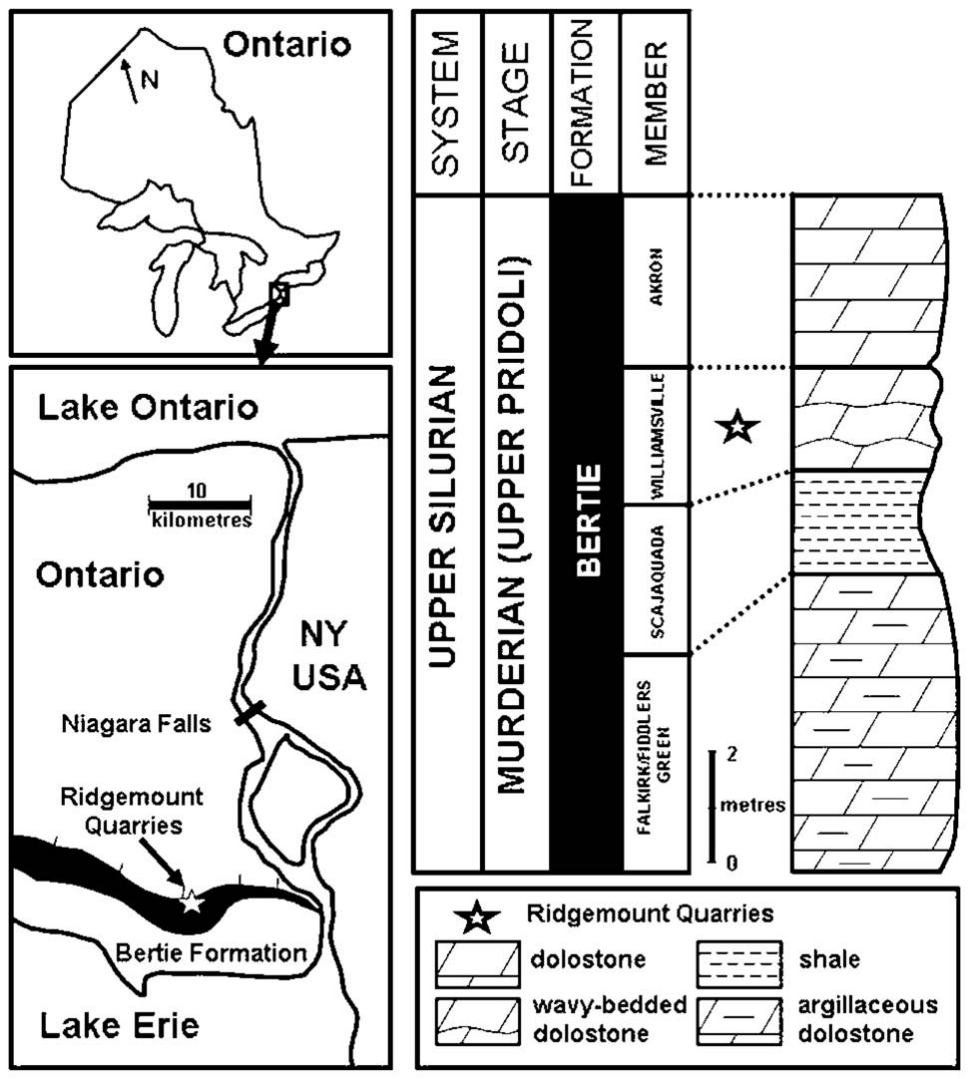
Locality map and stratigraphic column. Left, location of Ridgemount Quarries (star) within the Bertie Formation subcrop belt, Niagara Peninsula, southern Ontario; Right, generalized stratigraphic section of the Bertie Formation in the Ridgemount Quarries indicating the position (star) of the Williamsville Member (after [11]).

A new specimen of *Nerepisacanthus denisoni*, consisting of part and counterpart, was recovered from the Williamsville Member of the Bertie Formation in the south pit of Ridgemount Quarries (Walker Aggregates Inc.), near Stevenville, Ontario (Figure 1). Sediments of the late Pridoli (Murderian) Bertie Formation were deposited on the paleosouthern side of the subsiding Algonquin Arch, flanking the northern rim of the Appalachian Foreland Basin of Laurentia [6]. The Fiddlers Green and Williamsville members of the Bertie Formation in Ontario represent the northwestern edge of a much broader outcrop belt of long-famous “waterlime” deposits that extends into east-central New York State. These massive cement-like to argillaceous dolostones originated in subtidal and intertidal lagoonal settings, under alternating hypersaline and brackish estuarine conditions. Eurypterids, including species of *Eurypterus*, *Dolichopterus* and *Acutiramus*, are the best known and most spectacular faunal elements of the Bertie Konservat-Lagerstätte [7], and although most specimens appear to represent moults, they are often exquisitely preserved as thin cuticular remnants [8], [9], [10]. Other arthropods include *Bunaia* (a “synziphosurine”), the phyllocarid *Ceratiocaris acuminata*, and an exceptionally rare naraoiid [11]. Undescribed conodonts are found occasionally in etched residues and on bedding plane surfaces in the formation. Both fertile (*Cooksonia* sp.) and sterile (*Hostinella* sp.) axes of early land plants have been documented, along with presumed non-calcified algae (*Inocaulis* sp.) [12]. Lingulide brachiopods, orthocone and brevicone cephalopods, and ostracods comprise the most common shelly components of the biota. Biostratigraphic evidence for the age of the Bertie comes mainly from studies bracketing the unit below and above [13].

No vertebrates have been recovered previously from the Bertie Formation in Ontario or New York State. Here we describe a single, almost complete representative of *Nerepisacanthus denisoni* that confirms the assignment of this species to the ischnacanthiform family Acritolepidae, based on a combination of features including dentigerous jaw bones, shallowly inserted laterally flattened fin spines ornamented with smooth longitudinal ridges, absence of prepelvic fin spines, body scales with superposed growth crowns formed of odontocytic mesodentine, and scales in the head/branchial region with polyodontode, appositional growth crowns.

## Results

### Description of the new specimen

**Figure 2:**
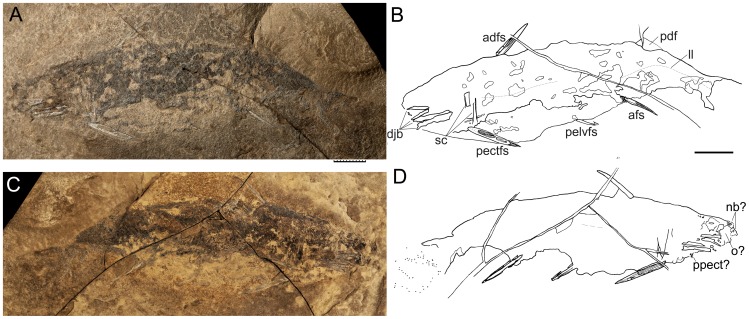
*Nerepisacanthus denisoni*, new specimen ROM64622 from the Bertie Formation. (A) part (right side, uppermost as deposited) photo and (B) outline drawing; (C) counterpart (left side, lowermost as preserved) photo and (D) outline drawing. Abbreviations: adfs = anterior dorsal fin spine; afs = anal fin spine; djb = dentigerous jaw bones; ll = lateral line; pdf = posterior dorsal fin; pectfs = pectoral fin spine; pelvfs = pelvic fin spine; sc = scapulocoracoid. Scale bars: 1 cm.

**Figure 3:**
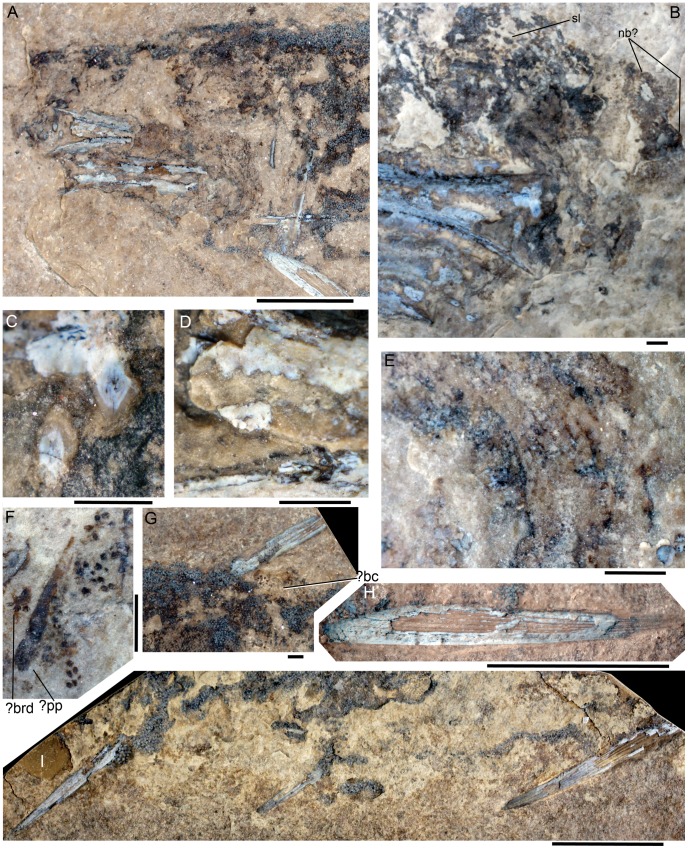
*Nerepisacanthus denisoni* ROM64622. (A) head and pectoral regions on part; (B) head region on counterpart; (C) natural sections through main cusps on lateral teeth of the upper set of jaws on the counterpart, and (D) subsidiary cusps on lateral tooth and section through a denticle on the median ridge of the left pair of dentigerous jaw bones, on the part; (E) gill region behind the lower set of jaws on the part; (F) remnants of possible prepectoral plate, below the jaws on the counterpart; (G) proximal half of the anterior dorsal fin spine on the part; (H) pectoral fin spine of the right side, on the part; (I) pectoral, pelvic and anal fin spines on the counterpart, head to right. Abbreviations: bc = basal cartilage; brd = branchial denticles; nb = nasal bones; o = orbit; pp = prepectoral plate; sl = sensory line. Scale bar: 1 cm A, H, I; 1 mm B–G.

Specimen ROM64622 of the acritolepid ischnacanthiform *Nerepisacanthus denisoni* [4] is approximately 112 mm long with maximum depth 29 mm, preserved as right (part) and left (counterpart) sides (Figure 2) by splitting of the laterally compressed fossil on its discovery. This splitting resulted in exposure of the bases of scales from each half of the body. Also, most of the larger dermal structures are split longitudinally, so that their surface morphology is only rarely visible. Fin spines preserved on the specimen are the anterior dorsal, paired pectoral, paired pelvic, and anal spines (Figure 2B). Two pairs of robust upper and lower dentigerous jaw bones, each ca. 12 mm long, are the most anterior robustly developed bony elements preserved (Figure 3A, B); the teeth ankylosed to these bones comprise a large upright triangular central cusp with several smaller cusps anterior and posterior (Figure 3C, D) forming a lateral tooth row, and a median or lingual ridge with ribbed denticles having a circular parabasal section (Figure 3D), similar to those of *Acritolepis ushakovi* [14] from the earliest Devonian of Severnaya Zemlya, Russia. Other dental elements (teeth, tooth whorls, dentition cones) cannot be clearly distinguished in the oral or branchial region, although fracture surfaces through teeth between the splayed anterior ends of the dentigerous jaw bones (Figure 3A) could be evidence of a symphysial tooth whorl. Gill structures on the right side are preserved as curved impressions with small fractured denticles in situ (Figure 3E), lacking any mineralization of the branchial arches. The branchial region is relatively short at ca. 8 mm long, shorter than the mouth region based on the length of the dentigerous jaw bones. Dark stains dorsal and anterodorsal to the jaw bones could represent remnants of soft tissue, eyes, cartilaginous structures and very thin dermal elements, but no structures are distinguishable that are comparable with neurocranial elements recognized in *Acanthodes*, the only acanthodian for which the whole cartilaginous skeleton is mineralized and has been frequently described (e.g. [15], [2]). Remnants of jaw cartilages are possibly represented by translucent calcifications next to the dentigerous jaw bones (Figure 3B). Other than these bones, the only ossified elements larger than scales distinguishable in the head region are a pair of poorly preserved, wide ring-shaped structures ornamented with radiating rows of small tubercles (Figure 3B) which are the anteriormost elements on the rostrum.

The pectoral girdle comprises only perichondrally ossified scapulocoracoids and pectoral fin spines, with no admedian plates or spines visible (Figure 3A). The scapulocoracoids are 12 mm high, and have a high thin-walled ‘half-pipe’ bone shaft that widens slightly towards the base, showing equal curvature along both the anterior and posterior edges. The shaft is ossified on the medial face (contra [4], which wrongly stated the lateral face of the scapulocoracoid was ossified). The fracture surface through an elongate bony element ca. 3 mm long, next to scattered tubercles or scales and a very thin bone, is exposed below the jaw bones (Figure 3F). These features are tentatively interpreted as a longitudinal section through a prepectoral plate.

All fin spines are straight and laterally flattened, with a shallow insertion, and widest ridges along the anterior and posterior edges. Pectoral fin spines (Figure 3H, I) are the largest, at 21 mm long, with seven longitudinal ridges per side and a very short insertion area lacking ornament ridges. The anterior dorsal fin spine is 16 mm long (Figure 3G), pelvic fin spines are 7 mm long, and the anal fin spine is 11 mm long (Figure 3I); all spines have five ridges per side. The anterior dorsal spine possibly has a basal cartilage (Figures 2A, 3G). The posterior dorsal fin spine is missing, but impressions of small scales of the posterior dorsal fin web are preserved behind a crack in the part ( = right side), slightly posterior to the level of the anal fin spine (Figure 2A, B).

**Figure 4:**
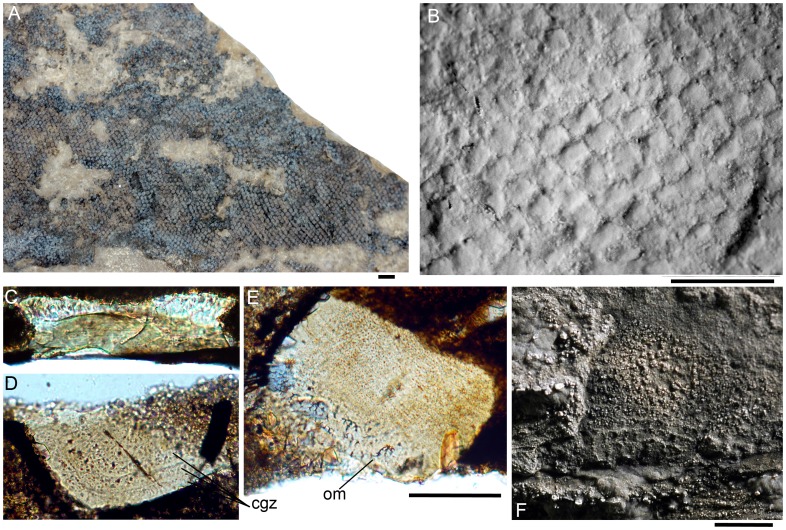
*Nerepisacanthus denisoni* ROM64622, squamation. (A) mid-flank on part, below anterior dorsal fin spine, showing lateral line position; (B) patch of scales on counterpart with crowns exposed midflank (anterior to top), whitened with magnesium oxide; (C) vertical thin section of midflank scale; (D) horizontal section through the anterior half of a scale crown; (E) oblique/horizontal section of midflank scale, through crown and base; (F) acid-etched squamation over the jaw joint on the counterpart, whitened. Abbreviations: cgz = crown growth zones; om = odontocytic mesodentine. Scale bar: 1 mm A, B, F; 0.1 mm C–E.

*Nerepisacanthus* has two types of scales, ‘classical’ acanthodian-type scales with superposed growth crowns and bone bases over the body and fins, and also scales in the cheek region which have appositional growth zones forming the crown. The body scales form diagonal rows over the flanks, as is the usual layout for acanthodians and some other micromerically scaled gnathostomes. A short length of a sensory line on the head is discernible as a row of parallel elongate scales posterodorsal to the dentigerous jaw bones (Figure 3B). The position of the lateral line is inferred from a slight change in the orientation of scale rows and/or a low ridge along the body (Figure 4A), as the scales bordering these lines do not differ from normal flank scales. Tail shape and squamation pattern is not clear, with only scattered scales preserved posterior to the caudal peduncle (Figure 2), although the scales in zone Z2 [16], along the dorsal edge of the fin, have remained intact (Figure 4B). Flank scale crowns are flat with only short weakly developed ridges along the anterior edge (Figure 4B). The scale neck and base are of similar maximum height, with the lower surface of the base moderately convex. The scales show minimal overlap of neighbouring scales (Figure 4B). Thin sections of the scales show typical acritolepid histological structure [14], with odontocytic mesodentine (i.e. a network of dentine tubules with lacunal widenings, sensu [14]) forming four or five superposed crown growth zones, and bone cell lacunae scattered through the base, which forms a low cone extending towards the centre of the crown (Figure 4C–E). No vascular canals are visible in the thin sections, but as histological preservation is rather poor their absence is probable but not certain.

Most of the head region shows only remnants of a scattered dermal cover of scales and tesserae; their surface morphology is not generally exposed, but their size is equivalent to that of normal body scales, other than the larger scales lining a sensory line (Figure 3B). Squamation in the cheek area (Figures 3A, 4F) – over the jaw joint and the front of the branchial region – comprises low, poorly preserved scales which show no evidence of superpositional crown growth zones, and differ distinctively from the body squamation. Such scales were also observed in the type specimen of *Nerepisacanthus denisoni*, with both the type and the new specimen showing a sharp boundary between normal body scales and the areal-growth polyodontode crowned scales. Unlike the flank scales, the polyodontode scales are oriented dorsoventrally, a feature now noted on the holotype but not recognized in the original description [4]. Unfortunately their original crown surface is not clearly visible on any of the scales or their impressions on ROM64622, as no whole polyodontode scales were preserved intact and pyritic infilling of individual odontodes remained after etching with 10% HCl, obscuring impressions of the crown surfaces (Figure 4F). Scales and tesserae with a similar growth pattern are found in the head and branchial region of Lower Devonian (Lochkovian) *Acritolepis* spp. from Severnaya Zemlya [14]. However, these scale forms in *Acritolepis*, like typical body scales of the genus, have moderately convex bases, and low necks, whereas those in ROM64622 are extremely thin.

## Discussion

**Figure 5:**
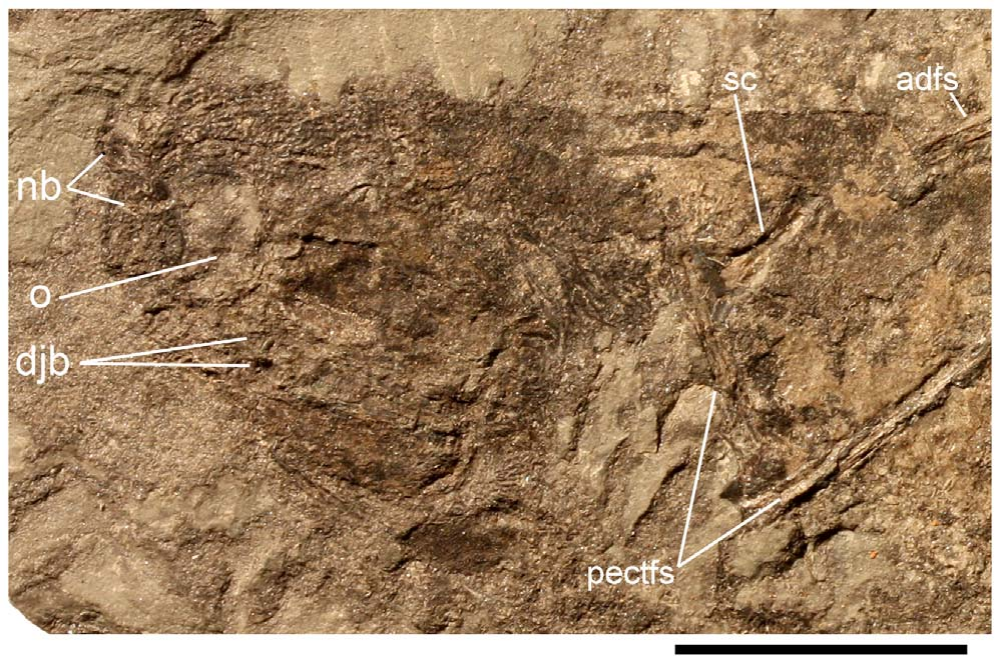
*Ischnacanthus gracilis* specimen NMS G.1891.92.265 from Tillywhandland Quarry, Forfarshire, Scotland. Articulated, laterally compressed fish with nasal bones (nb) preserved as the anteriormost dermal structures, in front of the orbit (o) and the dentigerous jaw bones (djb). Other abbreviations: adfs = anterior dorsal fin spine; pectfs = pectoral fin spine; sc = scapulocoracoid. Scale bar: 1 cm.

The new Bertie Formation vertebrate shows the same features described in the partial articulated type specimen of *Nerepisacanthus denisoni*: straight laterally flattened fin spines ornamented with smooth longitudinal ridges which are wide along the anterior and posterior edges and narrow between; thin walled high scapula shafts, unossified laterally, on the short-based scapulocoracoid; body scales with superposed growth zones formed of odontocytic mesodentine; scales anterior to the scapulocoracoid with polyodontode areal-growth crowns. The latter scales are oriented dorsoventrally in both the type specimen and the new Bertie fish, whereas flank scales are oriented anteroposterially. In addition, the new specimen also confirms that *N. denisoni* has dentigerous jaw bones, as inferred from one of the disarticulated type specimens [4], supporting its assignment to the family Acritolepidae [17] in the order Ischnacanthiformes [4]. ROM64622 is a smaller fish than the type specimen ROM1252; the anterior dorsal fin spine (the only spine preserved) on the type is nearly 30 mm long, whereas the same spine on ROM64622 is 16 mm long. The new specimen could be a juvenile, as the dermal head scales and tesserae appear sparsely developed [18], [19]. The ring-shaped ornamented plates are an interesting feature, as comparable plates have not previously been described in any articulated ischnacanthiforms. However, the head region is poorly known in *Acritolepis* and *Poracanthodes*, hampering comparison of *Nerepisacanthus* with other acritolepids and poracanthodids. The ornament is very similar to that of sclerotic plates in *Euthacanthus macnicoli* and *Climatius reticulatus* [20] and diverse plates near the mouth in *Parexus recurvus* [21], but those plates are very robust. Ischnacanthiforms *Ischnacanthus gracilis* and *Poracanthodes menneri* lack sclerotic plates [3], [22], and this is presumed to be a derived condition because stem gnathostomes and climatiid and acanthodiform acanthodians have sclerotic rings [20]. Given the rostral position of the structures, by comparison with better preserved acanthodians they are probably nasal bones – rare specimens of *Ischnacanthus gracilis* from the Lower Old Red Sandstone of Scotland have ornamented nasal bones in this position (Figure 5).

The chondrichthyan-like scales in *Acritolepis*, presumed to be from the head or branchial region, have overlapping appositional and areal crown growth zones, each with wide basal pulp cavities [14]. As none of the *Acritolepis* specimens have the head region well preserved, the original position of the scales is not known, however some scales [14] resemble those that edge sensory lines on the head in *Ischnacanthus gracilis* [23]. The elongate scales edging the dorsal sensory line on the head of *Nerepisacanthus* (Figure 3B) also appear comparable to these scales. None of the chondrichthyan-like scales in *Acritolepis* exactly resemble those in *Nerepisacanthus*, which compare closely with cladodont-type chondrichthyan scales [24], [25].

**Figure 6:**
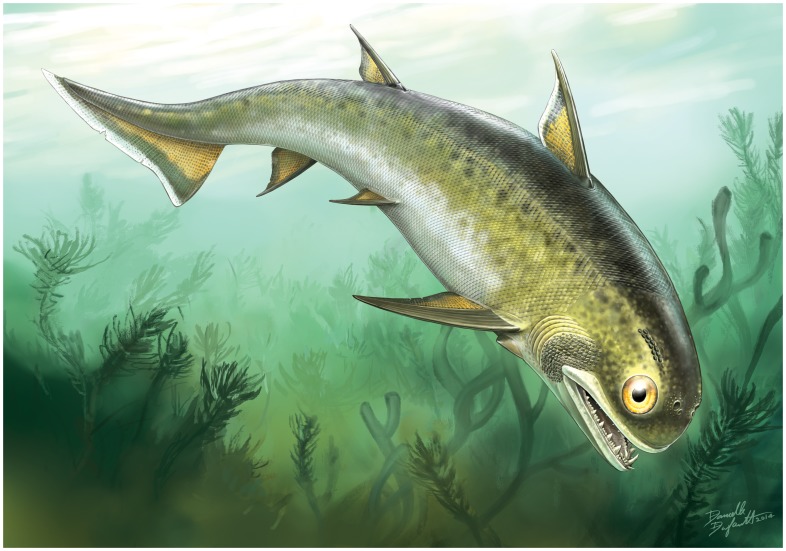
Reconstruction of *Nerepisacanthus denisoni*. Dynamic depiction by Danielle Dufault (ROM) of the fish swimming above algae in a lagoonal setting representing the Bertie Konservat-Lagerstätte paleoenvironment.

*Nerepisacanthus denisoni* (Figure 6) has the same suite of macrodermal elements (notably, the dentigerous jaw bones) as in articulated poracanthodid and ischnacanthid ischnacanthiforms of earliest Devonian age, exemplified by *Ischnacanthus gracilis*, *Poracanthodes menneri* [22] and unnamed ischnacanthids from the MOTH (Man-on-the-Hill) locality in the Northwest Territories, Canada [26]. Scale morphology and histology, however, show that *Nerepisacanthus* is an acritolepid ischnacanthiform [17], [4]. The younger type genus *Acritolepis* differs from poracanthodids and ischnacanthids in having paired triangular prepectoral plates. It is likely that *Nerepisacanthus* also has such plates; traces of a structure of the size and shape expected for this element (by comparison with the plates of *Acritolepis*) are preserved on ROM64622B (Figure 3F).

An important character shared by all acritolepids is the co-occurrence on the same animal of ‘classic’ acanthodian scales showing superposed, onionskin-type crown growth zones as well as polyodontode crowned scales which, if found as isolated remains, would previously have been assigned to cladodont chondrichthyans. Notably, one of the most recent analysis of early gnathostomes [27] has acanthodians and chondrichthyans forming a monophyletic sister group to all other gnathostomes, but with acanthodians as a paraphyletic assemblage on the chondrichthyan stem, and the most recent cladistic analysis [28] shows acanthodians as the monophyletic sister group to Chondrichthyes. Perhaps just as the Mesozoic dinosaurs have birds as an extant lineage, the Paleozoic acanthodians (previously considered to have died out in the Permian) have extant descendants – the sharks swimming in our oceans. ROM64622 is an exciting find, as the first vertebrate ever found in the Bertie Formation, but it is also a pivotal specimen, showing that *Nerepisacanthus denisoni* and the other acritolepids exhibit a suite of characters straddling the ‘fence’ between the putative stem chondrichthyan and stem osteichthyan lineages of some recent cladistic analyses [1], [2]. Future cladistic analyses incorporating *Nerepisacanthus* and updated data (including several articles in press) on other acanthodians used in previous publications could help clarify early gnathostome relationships.

## Materials and Methods

The specimen ROM 64622 A & B (part & counterpart) is reposited in the collection of the Royal Ontario Museum (ROM), Toronto, Canada. No permits were required for the described study. The fossil detailed in this research was legally collected from a commercial aggregate quarry with access granted by the quarry operators. There is no provincial legislation prohibiting the private collection or ownership of fossils in Ontario. The specimen in question was generously donated by the collector to ROM, which is an agency of the Government of Ontario and the only Ontario institution with a de facto mandate to acquire, study, and preserve the palaeontological heritage of the province.

Macro photographs were with several imaging systems including a Canon 6D DSLR camera mounting a Canon EF 100 mm f/2.8 Macro USM lens under bidirectional halogen lighting (Figure 2A, C), Nikon D70s with Micro Nikkor 55 and 105 mm lenses (Figure 4F with the specimen whitened with ammonium chloride sublimate); Foveon Camera Sigma SD10 DSLR with 50 mm F2.8 Sigma EX Macro Lens SA mount (Figure 3A, G); Panasonic DMC-G10 or G11 with Konica Hexanon 50 mm F1.7 AR mount + Bellows/extension tubes (Figure 3B–D); Olympus E-PL1 with Konica Hexanon 50 mm F1.7 AR mount + Bellows/extension tubes (Figure 3E, H); Sony NEX-3 with 50 mm F2.8 Sigma EX Macro Lens PK mount + Bellows/extension tubes (Figures 3F, I, 4A); the latter four systems photographed the specimen under water. A small area of cheek scales on the counterpart were covered with a few drops of 10% hydrochloric acid for 30 secs, then rinsed with water, in an attempt to dissolve them and expose the scale crown impressions. The fragment on the counterpart with scale crowns exposed was whitened with magnesium oxide and photographed using an Olympus SX50 microscope and DP12 imaging system. Thin sections were ground by hand using fine grade wet and dry sandpaper, and photographed using an Olympus BX50 transmission microscope and DP12 imaging system. Figures were prepared using Adobe Photoshop CS4.
